# Infant Mortality Related to NO_2_ and PM Exposure: Systematic Review and Meta-Analysis

**DOI:** 10.3390/ijerph17082623

**Published:** 2020-04-11

**Authors:** Wahida Kihal-Talantikite, Guadalupe Perez Marchetta, Séverine Deguen

**Affiliations:** 1 LIVE UMR 7362 CNRS (Laboratoire Image Ville Environnement), University of Strasbourg, 6700 Strasbourg, France; 2 EHESP School of Public Health, 35043 Rennes, France; guadalupe.perezmarchetta@eleve.ehesp.fr (G.P.M.); severine.deguen@ehesp.fr (S.D.); 3 Department of Social Epidemiology, INSERM, Institut Pierre Louis d’Epidémiologie et de Santé Publique (UMRS 1136), Sorbonne Universités, UPMC Univ Paris 06, 75646 Paris, France

**Keywords:** systematic review, meta-analysis, infant mortality, exposure, air pollution, PM, NO_2_

## Abstract

*Background*: We conducted this systematic review and meta-analysis to address the crucial public health issue of the suspected association between air pollution exposure during pregnancy and the risk of infant mortality. *Methods*: We searched on MEDLINE ^®^ databases among articles published until February, 2019 for case-control, cohort, and ecological studies assessing the association between maternal exposure to Nitrogen Dioxide (NO_2_) or Particular matter (PM) and the risk of infant mortality including infant, neonatal, and post-neonatal mortality for all-and specific-causes as well. Study-specific risk estimates were pooled according to random-effect and fixed-effect models. *Results*: Twenty-four articles were included in the systematic review and 14 of the studies were taken into account in the meta-analysis. We conducted the meta-analysis for six combinations of air pollutants and infant death when at least four studies were available for the same combination. Our systematic review has revealed that the majority of studies concluded that death risk increased with increased exposure to air pollution including PM_10_, PM_2.5_, and NO_2_. Our meta-analysis confirms that the risk of post-neonatal mortality all-causes for short-term exposure to PM_10_ increased significantly (pooled-OR = 1.013, 95% CI (1.002, 1.025). When focusing on respiratory-causes, the risk of post-neonatal death related to long-term exposure to PM_10_ reached a pooled-OR = 1.134, 95% CI (1.011, 1.271). Regarding Sudden Infant Death Syndrome (SIDS), the risk also increased significantly: pooled-OR = 1.045, 95% CI (1.01, 1.08) per 10 µg/m^3^), but no specific gestational windows of exposure were identified. *Conclusion*: In spite of a few number of epidemiological studies selected in the present literature review, our finding is in favor of a significant increase of infant death with the increase of air pollution exposure during either the pregnancy period or the first year of a newborn’s life. Our findings have to be interpreted with caution due to weaknesses that could affect the strength of the associations and then the formulation of accurate conclusions. Future studies are called to overcome these limitations; in particular, (i) the definition of infant adverse outcome, (ii) exposure assessment, and (iii) critical windows of exposure, which could affect the strength of association.

## 1. Introduction

Despite considerable improvement in the prevention, management, and regulation of air pollution, it remains a leading environmental health issue worldwide and has been identified as a health priority on the sustainable development agenda [1]. Having clean air to breathe is a fundamental requirement for human health and well-being. While the increased risk to health of air pollution is relatively low in comparison with other risk factors, the total number of people affected is significant. According to the Organisation for Economic Cooperation and Development, air pollution is known to be the main environmental cause of premature death (2014) [2]. Environmental policies aiming to tackle the air pollution issue have proved to be effective, having health benefits. However, using a recent air quality model, the World Health Organisation (WHO) has calculated that 92% of the population lives in places where air quality levels exceed WHO limits [3]. Certain groups within the population are known to be more vulnerable to the health effects of air pollution exposure, and one such group is newborns and infants because of their overall physiological immaturity [4,5].

While the findings of epidemiological studies investigating risk factors are essential in public health, quantitative Health Impact Assessments (HIAs) are key to public policy regulatory and decision-making processes because they provide valuable information on future health concerns related to any potential intervention. HIA methodology calls upon a diverse set of data sources, including the dose-response function which indicates the expected change in a given outcome per unit change of pollutant deriving from epidemiological studies that assess the relative risk associated with the observed or modelled exposure [6]. This relative risk may come from meta-analyses providing pooled estimates. The benefits of a meta-analysis are that it offers relative risk estimation within a specific vulnerable population as well as a better fit with the geographical context of exposure.

In previous years, there has been an increase in the number of studies investigating links between infant mortality and air pollution. The potential impact of air pollution exposures on infant mortality has already been reviewed in 2004; that study suggested a stronger association between particulate air pollution and some causes of infant death [7]. Since then, several recent studies have been published [8,9,10]. In this context, an updated literature synthesis might tell us whether the current epidemiological evidence favours an association between infant mortality and air pollution, with a view to suggesting future directions for research.

## 2. Material and Methods

### 2.1. Search Strategy

A systematic literature search was conducted using the PubMed platform which provides access to the MEDLINE^®^ database and Academic Search Complete databases, among articles published up until February, 2019. The search strategy followed the PRISMA (Preferred Reporting Items for Systematic reviews and Meta-Analyses) guidelines [11] and was performed with the following keywords found in articles’ titles and/or abstracts:

“Air pollution” OR “Air pollutant” OR “Air Pollutants” or “outdoor Pollution” OR “Particular matter” OR “PM25” OR “PM10” OR “Black Emission” OR “Black Carbon” OR “Nitrogen Dioxide” AND “Infant Mortality” OR “Infant Mortalities” OR “Infant Death” OR “Child Mortality” OR “Child Mortalities” OR “Child Death” OR “Child Deaths” OR “Under-one Mortality” OR “Under-one Mortalities” OR “Under-one Death” OR “Under-one Deaths” OR “Neonatal Mortality” OR “Neonatal Mortalities” OR “Neonatal Death” OR “Neonatal Deaths”.

### 2.2. Selection of Studies

**Figure 1:**
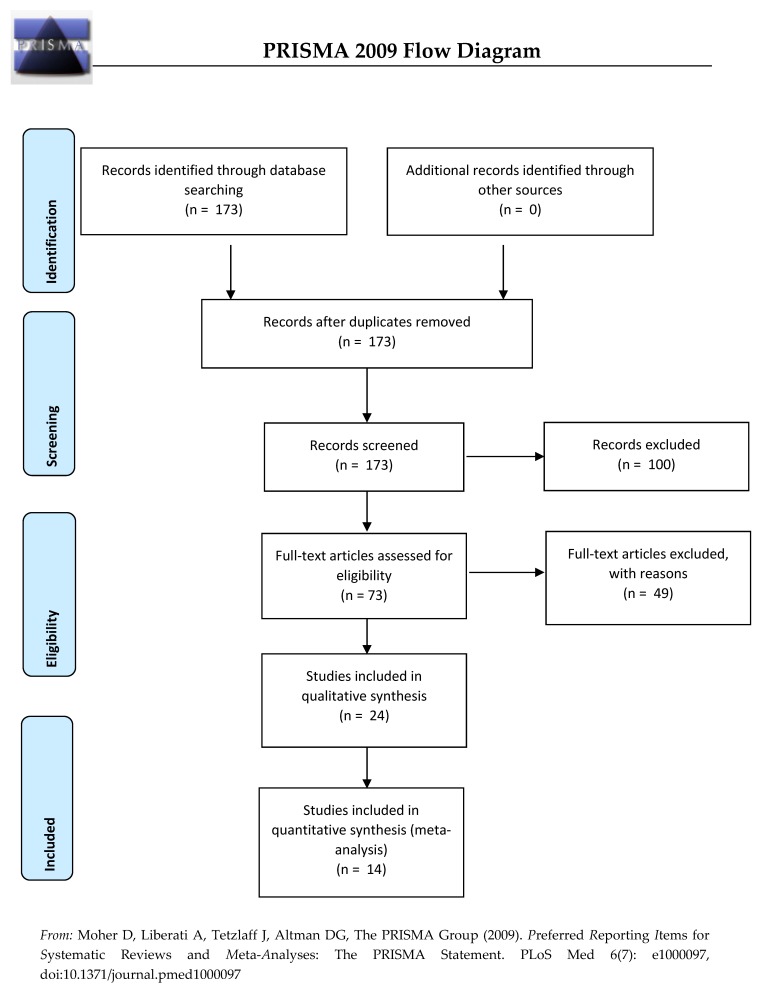
Flow diagram for the inclusion and exclusion of studies.

In line with PRISMA recommendations, Figure 1 summarizes the different stages of the selection process.

In the first stage, the inclusion criteria were peer-reviewed papers written in English and articles published after 2000 without restriction on geographical location and human studies. We restricted our systematic review on the children aged under 1 year. Papers presenting non-original studies (e.g., comments, systematic reviews, meta-analysis, reports, case reports, animal and mechanistic studies, and biological experiments) were ultimately excluded. Using these criteria, 173 of the 280 articles published were selected for inclusion.

In the second stage, titles and abstracts of the 173 articles were screened. A total of 100 studies were then excluded when they:

*(i)*  Investigated adult mortality, other pregnancy, birth, infant, or child outcomes
*(ii)*  Considered indoor air pollution, smoking exposure, or were traffic-related (industrial plants, all types of wastes, cooking and biomass fuels consumption, ambient conditions, other environmental exposures)
*(iii)*  Dealt with other outdoor air pollutants measured (not including PM_2.5_, PM_10_, or NO_2_)

In the third stage, full manuscripts of the remaining 73 articles (of the 173 initially selected) were read thoroughly by two independent experts; 24 studies were retained if:

*(i)*  Studies investigated the death among children aged between 0 and 1 years old.
*(ii)*  Studies considered only the air exposure traffic related.
*(iii)*  The outdoor air pollutants measured included at least PM_2.5_, PM_10_, or NO_2_, the three pollutants of interest.
*(iv)*  Studies were original studies quantifying the relationship between infant mortality and outdoor air exposure related traffic (not non-original studies, opinion articles, comments, critical, narrative, and systematic reviews and meta-analysis, Global Burden of Disease studies, HIAs, environmental health indicators, risk assessments)

Ultimately, a total of 24 articles met the inclusion criteria for the systematic literature review.

In order to perform a meta-analysis, studies were excluded where there was:

*(i)*  no measure of association
*(ii)*  a measure of exposure not expressed as a pollutant concentration (for instance: exposed/not exposed)
*(iii)*  when either outcome (neonatal mortality) or exposure (NOx in the summer season) was not pertinent for the meta-analysis

Also, meta-analysis was not performed where measures of association between a given outcome and a pollutant were available for at least four studies.

Hence, of the 24 articles included in this systematic literature review, 10 did not meet the inclusion criteria for the meta-analysis. In the end, 14 articles were included in the meta-analysis.

### 2.3. Data Extraction

For each study, we extracted and reported the following information in several tables:

*(i)*  General information: first author’s name, date of study, and country of origin,
*(ii)*  Main study characteristics: study design, spatial unit, statistical methods, population definition, main findings (related to infant outcomes and PM_10_, PM_2.5_, and NO_2_ only),
*(iii)*  Participant characteristics: information on confounders and exposure measures,
*(iv)*  Outcome measures: outcomes classification, definition, and source,
*(v)*  Measures of association were extracted including Hazard Ratios (HRs), Odds Ratios (ORs), Relative Risks (RRs), and other metrics measuring the strength of association between mortality and exposure to different pollutants including PM_10_, PM_2.5_, and NO_2_. Where several measures of association were available, we reported those taken from the fully adjusted models.

### 2.4. Meta-Analysis

The pooled estimate between exposure to air pollution and mortality was computed only where at least four studies were available. All risk estimates were expressed as unit risks corresponding to an increase of 10 µg/m^3^. The combined effect was obtained from a fixed or random model based on the Cochran Q-test, the I-square statistic, and the associated *p*-value. Where the Cochran Q-test revealed significant heterogeneity between studies, a random model was implemented; conversely, a fixed model was applied where the Q-test was not significant. The I-square (I^2^) indicator quantifies the level of heterogeneity between studies. Where the value varies between 25% and 50%, 50% and 75%, and >75%, this corresponds to a low, medium, and high level of heterogeneity, respectively. Forest plots were created to represent the combined risk estimates. All statistical analyses was performed using STATA 11.

#### 2.4.1. Publication Bias

Funnel plots, which present effect sizes plotted against their standard errors, were used to assess for potential publication bias. The asymmetry of the funnel plot is an indication of publication bias which can be confirmed by applying the begg‘s rank test for small-study effects. This test examines the correlation between the effect size and their corresponding sampling variances, with a strong value of correlation meaning a publication bias.

#### 2.4.2. Sensitivity Analysis

In addition, we also evaluated the influence of each individual study on the overall meta-analysis estimate; we implemented several meta-analyses in which the meta-risk is re-estimated, omitting each study in turn (we used the metaninf function in STATA software (StataCorp, College Station, TX, USA). While there is no formal statistical test to prove that such a study should, or should not, be removed from the analysis, we followed two general guidelines to assess the influence of a given study. We analyzed if the point estimate of this omitted analysis lies outside the confidence interval of the combined analysis and second, if the omitted study excessively influenced the significance of the combined risk.

## 3. Results

### 3.1. Main Characteristics of the Studies

#### 3.1.1. General Description

Table 1 provides the characteristics of all the studies reviewed by year of publication, type of study design, infant mortality outcome, exposure assessment, and major findings and conclusions.

Since 2000, 24 studies (covering more than 400,000 infant deaths) had been published to estimate the association between outcomes and exposure to three ambient pollutants: PM_2.5_, PM_10_, and NO_2_. Of these, infant mortality all causes, post-neonatal infant mortality all causes, respiratory causes of infant and post-neonatal mortality, and sudden infant death syndrome were investigated (Table 1). Only 14 of the studies were eligible for meta-analyses.

#### 3.1.2. Study Design and Location

Most of the studies were conducted in the United-States (in both the north and south) [8,10,12,13,14,15,16,17,18,19,20,21]. There were also 6 studies conducted in Europe and the UK [9,22,23,24,25], 7 studies conducted in Asia [26,27,28,29,30,31], and a single study conducted in Africa [32]. Our systematic review is grouped by study design: a majority are ecological studies [9,12,19,20,23,24,31]; others are case crossover studies [8,18,22,25,26,28,29,30], case-control studies [15,16,17], cohort, and cross sectional studies [10,13,14,27,32].

#### 3.1.3. Cases Definition and Data Sources

While many studies investigated only the overall group of infant mortality [9,10,15,21,23,24,25,26,32], post-neonatal [8,9,13,21,25,26,27,28], and neonatal death [9,10,15,21,23,25,26], others explored specific cause of death, mainly by respiratory causes [8,10,12,13,14,15,16,18,21,25,26,27,31] and Sudden Infant Death Syndrome (SIDS) [13,15,17,19,21,22,26,27]. Several studies stratified their analysis on an additional covariate. In 2011, Scheers et al. [25] analyzed the risk of death among a subgroup of newborns according to their birth weight (low birth weight versus normal birth weight) as well as the studies of Son et al. in 2011 [27] and Woodruff et al. in 2008 and 2006 [13,16]. Other authors stratified their analysis according to birth term (preterm versus at term) [15]. The definition of infant mortality by causes was relatively homogeneous across studies (see Table S1). Among studies investigating respiratory causes of death or SIDS, all but four (which did not give any precision [10,17,19,21]) based their outcome definition on the International Classification of Disease 9 and 10 (ICD 9–10). Databases were drawn mainly from birth and death certificates as well as from institutes such as the Institute of National Health Statistics or the Ministry of Health (see Table S1).

### 3.2. Air Pollution Exposure Assessment

In Table 2, the study’s results were structured by approaches that have been used to assess the level of residential exposure. Table 3 describes the different definitions of exposure windows considered in the 24 studies included in the systematic review.

#### 3.2.1. Pollutants of Interest

Most studies investigated the effects of a single air pollutant, although a few looked at the effects of multiple pollutants [13,15,19,20]. The most frequently analysed air pollutants were PM_10_, PM_2.5_, NO_2_, CO, O_3_, NO_2_, and SO_2_ [9,19,20,21,22,28,29,30], although others studied considered more specific pollutants such as PM_10-2.5_ and TSP [27], PM_7-2.5_ and SPM [26]. The number of air pollutants included in studies to investigate the health consequences of exposure varied between 1 and 5: most analysed the effects of PM_10_, NO_2_, CO, O_3_, and SO_2_ [9,19,20,21,22,28,29,30], though others considered only PM_10_, CO, O_3_, and SO_2_ [10,13] or PM_10_, CO, O_3_, and NO_2_ [15]. Few studies focused on the effects of certain pollutants, namely PM_10_, PM_2.5_, and CO [14], or indeed the effects of just two pollutants, PM_10_ and O_3_ [8,12,18] or NO_2_ and CO [17]. Five studies considered a single pollutant, namely PM_2.5_ [16,32], PM_10_ [25], or NO_2_ [23,24].

#### 3.2.2. Exposure Definition

All studies considered air pollution data from monitoring stations, except four studies [23,24,25,32] which based their measures of exposure on a modelling approach (Table 2). Whichever methodology was applied to characterize residential exposure, most often it was on a daily basis (except in four studies [21,23,24,32], which examined annual indicators). For all air pollutants (PM_10_, PM_2.5_, CO, NO_2_, and SO_2_), the authors most often used the daily (24 h) average, except in two studies which selected the maximum daily concentrations observed as the indicator of exposure [10,17]. Daily average O_3_ exposure was used by all studies bar three [8,12,18], which estimated the daily maximum of the eight-hour moving average as the exposure indicator.

The description of all studies included in the systematic review (n = 24) by approaches used to assess the residential exposure measures and Level exposure assigned to the population is shown in Table 3.

#### 3.2.3. Window of Exposure

Table 3 revealed that both short- and long-term exposure to air pollution were used to investigate the relationship between residential exposure and infant mortality; short-term exposures were the most commonly explored exposure windows using various indicators that include daily exposure and cumulative exposure. Moreover, some studies chose not to focus on a particular critical window of exposure, instead measuring annual average pollutant concentrations at dwelling [21,23,24].

### 3.3. Meta Analysis

#### 3.3.1. Main Characteristics

Our meta-analysis was conducted for 6 combinations between one air pollutant and one infant death when at least four studies were available for the same combination. More precisely, the 6 combinations were post-neonatal death all-causes related with (1) NO_2_ exposure and (2) PM_10_ exposure, post-neonatal death due to respiratory causes related with (3) PM_2.5_ and with (4) PM_10_ exposure, as well as sudden infant death syndrome (SIDS) related with (5) PM_2.5_ and with (6) PM_10_ exposure. All the measures of the association of the studies included in the meta-analysis are detailed in Table S2.

Where possible, stratified analyses have been performed in order to differentiate the health effect related to short- and long-term and daily or cumulative exposure. In all, 12 meta-analyses were implemented: of these, heterogeneity (Q-test) tests indicated eight meta-analyses with high I^2^ values (above or close to 50%) for which random effects models were applied (for the other four combinations, fixed models were used). Heterogeneity varied from 0% to 96.5%, indicating that measurement methods, sample properties, and characteristics varied both among and within different studies.

#### 3.3.2. Specific Causes Death

##### Post-Neonatal Death All-Causes

**Figure 2:**
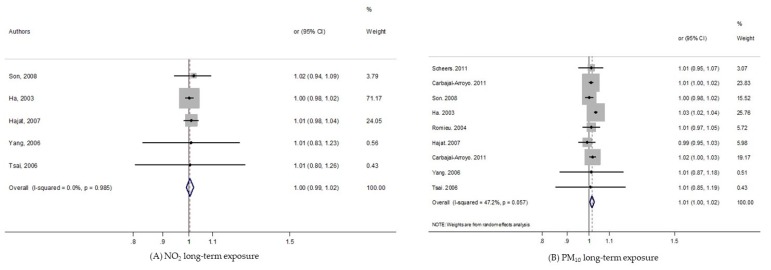
Forest plots for combinations of post-neonatal death all-causes and pollutant. The size of each square represents the weight that contributes to the combined effect, respectively for: (**A**) NO_2_; (**B**) PM_10_.

As shown in the Figure 2, we found no significant increase of pooled-OR for exposure to NO_2_, while it clearly became significant with short-term PM_10_ exposure: pooled-OR = 1.013, 95% CI (1.002, 1.025).

##### Respiratory Post-Neonatal Death

**Figure 3:**
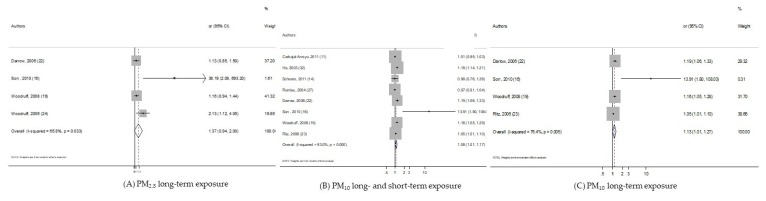
Forest plots for combinations of post-neonatal death Respiratory-causes and pollutant. The size of each square represents the weight that contributes to the combined effect, respectively for: (**A**) Long-term PM_2.5_; (**B**) long- and short-term PM_10_; (**C**) long-term PM_10_.

As shown in the Figure 3, the long-term exposure of PM_2.5_ on post-neonatal death due to respiratory causes was not statistically significant. While the overall analysis of PM_10_ exposure did reveal a significant increase in pooled-risk (pooled-OR = 1.082, 95% CI (1.005, 1.165)), the stratified analysis indicated that it only remained significant among studies considering long-term windows of exposure: OR = 1.134, 95% CI (1.011, 1.271).

##### Sudden Infant Death Syndrome

**Figure 4:**
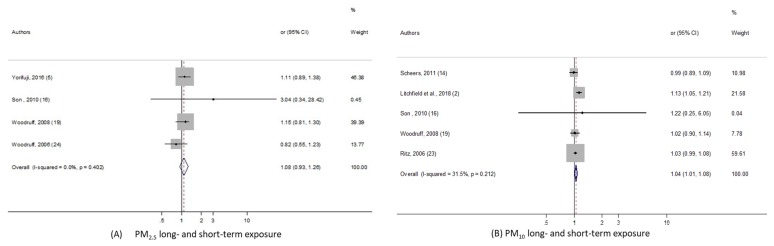
Forest plots for combinations of sudden infant death syndrome and pollutant. The size of each square represents the weight that contributes to the combined effect, respectively for: (**A**) Long- and short-term PM_2.5_; (**B**) long- and short-term PM_10_.

Regarding sudden infant death syndrome, 2 meta-analyses, with PM_10_ and PM_2.5_, were performed. As shown in the Figure 4, we found significant pooled-OR when considering PM_10_ exposure (pooled-OR = 1.045, 95% CI (1.01, 1.08) per 10 µg/m^3^); although when we kept only the three studies exploring the long term PM_10_ effect, the level of heterogeneity fell to 0%, yet the meta-risk was not significant at all: pooled-OR = 1.029, 95% CI (0.988, 1.072). In addition, the pooled-risk between SIDS and PM_2.5_ was not statistically significant.

#### 3.3.3. Publication Bias

Funnel plot and Begg’s rank tests were applied to determine whether there was publication bias. All funnel plots are in Figure S1. The results summarized in Table 4 present a low probability of publication bias, reporting a *p*-value for Begg’s rank test over 0.05, except for respiratory post-neonatal deaths for which borderline *p*-values were obtained.

#### 3.3.4. Sensitivity Analysis

Sensitivity analyses were performed to estimate the stability of our results by recalculating the pooled effects estimates after omitting one study each time (Table S3). For three meta analyses, we did not perform it due to too few numbers of studies. We found that the effect estimate of each 10 µg/m3 increase in NO_2_ and PM_10_ on post-neonatal death showed no significant change by removing one single study, suggesting that the combined results were relatively stable and reliable. Small variations were visible for respiratory post neonatal death and sudden death syndrome related to PM10 long- and short-term exposure; while point combined estimates were rather similar, the precision level of the confidence interval weakly decreased, leading to insignificant results with a lower limit of the 95% confidence interval less than but close to 1.

## 4. Discussion

### 4.1. Main Finding

Our systematic review has revealed that most studies conclude that there is an increased risk of infant death as a result of exposure to air pollution including PM_10_, PM_2.5_, and NO_2_. More precisely, our meta-analysis estimated a significant excess risk of post-neonatal mortality all-causes for short-term only exposure to PM_10_. We have also shown that the risk of respiratory post-neonatal death increased with a 10 µg/m^3^ increase in PM_10_ for long-term exposure specifically, as did the risk of SIDS with no specific gestational windows of exposure. In contrast, no significant excess risk of infant death was found regardless of pollutant or gestational window of exposure (including short- or long-term).

Taking into account the characteristics of the different studies (design, adjustment, definition of the outcomes....) (see S1, Table S4), these did not change the meta-risks estimated with the classical meta-analysis approach (data not shown).

These results could be partially explained by methodological limitations inherent to (i) definition of infant adverse outcome, (ii) exposure assessment and (iii) critical windows of exposure, which could affect the strength of the association. In addition, several inaccuracies and biases inherent to meta-analysis methods may bias cross-study comparisons and any conclusions drawn from them.

### 4.2. Outcome Data: Case Selection

We identified several pathways through which outcome data can lead to a bias in the measures of association. Firstly, outcome definition itself could constitute a source of uncertainty. The definitions of infant mortality used were (surprisingly) heterogeneous between studies, rendering comparisons difficult. For instance, although most of the studies have considered all deaths occurring among infants aged <1 year, few excluded neonatal mortality (death < 28 days) in order to consider the deaths most plausibly associated with air pollution [13,28] in terms of biological mechanisms. In addition, findings may be distorted as a result of some studies that were excluded, for instance, accidental [8,29,30,31] or external [15]. With regard to the definition of SID, two studies (26, 29) based their case definition on autopsies while other studies based the definition of SIDS cases on all unexplained deaths [21].

Another source of limitation lies in the health databases analyzed, which led the authors to collect different newborn and maternal characteristics. For instance, several studies investigated air pollution effects on infant death by cause [10,21,23,24], whereas others restricted their analysis to specific cause of death [12] or indeed considered all-causes of death due to lack of precise information [32].

Also, many studies ignored well-known risk factors for infant death such as gestational age, birth weight, and maternal age [9,10,12,18,20,22,23,24,25,26,32]. Furthermore, several other studies considered birth weight and/or gestational age as potential modifiers of the association between infant death and air pollution exposure. In order to do this, these studies stratified their analysis on birth weight by considering the infant death effect of air pollution exposure separately among the low and normal birth weight [21,27], or among preterm and normal term birth [25]. Two other studies combined birth weight and gestational age including only births beyond 44 weeks of gestation [13] or between weeks 37 and 44 [27].

### 4.3. Exposure Assessment

Different approaches for exposure assessment were implemented, and this may induce misclassification of exposure. Most of the studies used air pollution data from monitoring stations as a proxy for individual exposure. The main advantages of these databases relate to their easy accessibility and availability. However, their use presents several limitations, particularly when the objective is to quantify individual levels of exposure and investigate the health consequences of exposure.

The first of these limitations is related to the method used to convert concentration measures from monitoring stations to individual exposure: most of the studies either averaged air pollution concentrations from all monitoring stations covering the study area or selected a sub-sample of monitoring stations—just one, perhaps the one the closest to the dwelling [9,18,21,22,27,28,29,30]—while in others, no detail was given at all [8,12,13,14,19,20]. Many studies developed a methodology for identifying the nearest monitoring stations, then estimated the exposure level of the pregnant women [10,15,16,17,26]. For instance, one study defined the closest monitors at the zip/post code scale [17], whereas another quantified the maximum distance from the maternal dwelling to the monitoring station [16]. One study extended this procedure by using the inverse of the distance to nearby stations to weight measurement of the pollution estimate for each of the 56 municipalities in Mexico City [10]. In 2016, Yorifuji et al. considered air pollution data measured at a monitoring station located about 12 km from the central point of the ward’s spatial scale [26]. Another study combined identification of the nearest air monitoring station with the geographic features and wind flow patterns of the zip/post code at place of birth [15] (see Table 2). In addition, both the number of monitoring stations and the size of the study area vary between studies and this may increase the level of heterogeneity of air pollution measurement between studies. To be precise, the number of monitoring stations varied between a minimum of 5 [18] and a maximum of 27 [27,28]. There is also a risk that a small number of monitoring stations covering a large area may limit spatial representativeness of exposure, which may in turn introduce bias in assessment of the residential exposure of pregnant women. A further limitation comes from missing residential postal addresses; in such cases, the spatial unit chosen by the authors ranged from post-code level [22] to country level [27,28]. Misclassifications of exposure may result from the spatial unit used, with the largest spatial scale being less appropriate for the capture of fine spatial dispersion of air pollution concentrations.

Although data from monitoring stations is based on both national air quality requirements and guidelines and legislation that are compliant with approved methods [33], environmental modeling approaches now provide a higher level of spatial precision in exposure estimates than approaches based on routine monitoring station data. In our systematic review, two French studies used atmospheric dispersion modelling to estimate annual average NO_2_ at a census block level (9, 4), which was recognized as appropriate for the capture of spatial variabilities of air pollution. In 2011, Scheers et al. used a land use regression model to interpolate PM_10_ concentrations at a municipality level [25] (see Table 2). Only one study used satellite-based measurements of annual average PM2.5 concentrations at country level [32] (see Table 3). Environmental modelling is relatively cumbersome, labour-intensive, and computer-intensive, and also requires extensive data input; however, it is still held up as the gold standard for environmental and health impact assessment.

Lastly, regardless of which approach is chosen, exposure misclassification can also occur following changes in residential place during pregnancy. In general, studies are unable to take this limitation into account due to a lack of information about the residential mobility of pregnant women. However, residential mobility among pregnant women is not insignificant; in 2012, Bell et al. showed that the percentage of women who moved house during pregnancy ranged from 9% to 32%, with a median of 20% [34]. In addition to this residential mobility, it is even more difficult to estimate the daily mobility of pregnant women across the study area. No study included in the systematic review considered this important parameter, although some studies did suggest that pregnant women’s everyday mobility across the city would increase daily exposure [35].

The choice of pollutant used to describe exposure to air pollution is also crucial. Among the studies of our systematic review, few estimated possible multi-pollutant health effects [13,15,19,20]. Yet the fact that the health consequences of pollutant exposure does not result from a single pollutant is already well established; every day, everywhere, we are exposed to a cocktail of pollutants (including both indoor and outdoor air pollution) and new methodological developments are required in order to consider this issue and overcome method limitations.

### 4.4. Critical Windows of Exposure

Exposure misclassifications also depend on the definition of window of exposure. In our systematic review, two main approaches define the window of exposure in order to investigate the relationship between residential exposure and infant deaths: (i) short-term exposure (≤2 weeks) and (ii) long-term exposure (>2 weeks).

Even if we separately analyzed the effects of short- or long-term exposure, exposure heterogeneity may result from the various indicators implemented to measure the level of exposure. For instance, different indicators defining the daily exposure were identified: the day of the death (Lag0) [8,20,25,26,31], the day before death (Lag1) [8,18,22], or longer lags such as from lag 1 to lag 3 [18,22,25] or from lag 1 to lag 6 [22] (see Table 3). The studies that investigated short-term cumulative exposure also examined different windows of exposure including over 2 days (Lag0–2) [9,18,22,29,30], 3 days (Lag0–3) [8,12,17,18,19,25], 4 days (Lag0–4) [26], 6 days (Lag 0–6) [22], or over 7 days before death (Lag 0–7) [17]. More specific windows of exposure were also examined by Lin et al.; they considered exposure from two to seven days before death (Lag 2–7) [20]. Only one study focused on longer periods of exposure: 2 weeks before death [15] (see Table 3).

Regarding long-term exposure, two types of windows of exposure were identified (i) exposure during pregnancy and (ii) exposure of the newborn. In both cases, their exposure measures were based on cumulative exposure during a given period. During pregnancy, the studies measured exposure by trimester or during the entire period of pregnancy [27,32]. After birth, different windows of exposure were investigated including: the first month of life [15,17], the first 2 months before death [13,14,15], or the 6 months before death [15]. Larger windows of exposure were also examined—for instance, exposure during the first year of a newborn’s life [16,17,27,32] (see Table 3). Several other studies did not focus on a particular window of exposure and measured the annual average of pollutant concentrations at the residential place as a proxy of newborn exposure [21,23,24] (see Table 3).

### 4.5. Assessment of the Relation between Air Ambient Pollution and Infant Mortality

Our findings have to be interpreted with caution due to weaknesses that could affect the strength of the associations and then the formulation of accurate conclusions. In particular, the various confounding factors and the different sample size may lead to difficult between studies comparisons. Indeed, several studies adjusted for only meteorological characteristics (e.g., temperature, humidity, and seasonality) [28,29,30,31]. Some studies did not use any covariates [23,24,32], while others adjusted other studies adjusted on both baby and mother characteristics (maternal age, education, and marital status) and less often, on neighborhood characteristics, such as neighborhood socio-economic status [13,14,16,17,21,27].

Because of the lack of available information on dietary factors (such as folic acid supplementation, folic acid and vitamin intake during pregnancy), no study has adjusted risk estimates for these variables. An absence of systematic adjustment on common known confounders may affect the measure of association and thus, the comparison of all the risk estimates.

In addition, as any epidemiological study, the sample size may affect the statistical power: the higher the sample size, the higher the statistical power. Thus, in our study, we included studies with small sample sizes that provided imprecise estimates [36].

The features of the studies described above—such as study population, study design, sample size, the classification and definition of infant death, exposure assessment, and confounding factors—could all, independently or in combination, affect the quality of each study itself and, also, their comparison in our systematic review.

### 4.6. Strengths and Limitations

In addition to the limitations listed above, both our systematic review and our meta-analysis, like all studies, present their own strengths and limitations. Firstly, our work may suffer from study selection biases. Non-English publications of relevant articles may have been ignored. In addition, we cannot rule out the possibility that our systematic review, like most, could be impacted by publication bias. Indeed, unpublished results (including, in particular, results not statistically significant and grey literature, which is not available on open sources) may distort our meta-analysis findings towards the statistical significance of the risk estimates.

Also, the global level of air pollution in each country was not taken into account in our studies comparison, while we know that differences exist between countries. Thus, the health effect of a 10 µg/m^3^ increase in a pollutant could be measured in an area with a globally low level of air pollution or in an area with a high level. However, we had too few studies in our systematic review to stratify our analysis on the global level of air pollution. For similar reasons, it was not possible to perform a dose-response function analysis due to the low number of studies and the heterogeneity between them.

However, our review could form the basis for future research. Our systematic review was based on a large number of original studies and our meta-analysis presented six combinations of air pollutants and outcomes. We also detailed several sources of variability which may partially explain the observed measures of association. Future studies could be based on this analysis of limitations of the current body of research, which may provide inspiration for research agenda improvements.

### 4.7. Public Health Implication

Scientific works have been providing evidence of the health consequences of pollutants for a long time now. An increasing number of studies are now addressing the question of which policy strategies are needed to reduce exposure to environmental pollutants very early in life, before birth and sometimes also a few months before conception. Alongside this, attention on health impact assessment of air pollution has been on the rise in recent decades. The WHO recommends its use for both quantitative estimation of the current health effects attributable to air pollution and as a source of further evidence for public health action. According to the WHO, “*health impact assessment (HIA) is a practical approach used to judge the potential health effects of a policy, programme or project on a population, particularly on vulnerable or disadvantaged groups*” [37]. HIAs estimate the expected public health impact in the event that air pollution levels change to a given extent [38]. A crucial indicator required for quantification of the health burden of air pollution is the dose-response function, which is obtained from meta-analysis. This function indicates the expected change, on average, in a given outcome per unit change of pollutant. Our meta-analysis results provide pooled-risk for 6 combinations of air pollutants and infant death, which may provide the first step of the HIA. Because an HIA can estimate the human health impacts of current policy or implemented actions, it can become a useful tool for both policymakers and planners.

## Figures and Tables

**Table 1:** Main characteristics of the selected studies, ordered by year of publication.

Auteurs	Study Design, Period Location	Population Size	Outcomes	Pollutants	Statistical Methods	Confounders/Stratification	Main Findings
Lipfert et al., 2000 [21]	Cross-sectional study. 1990. USA.	13,041 infant deaths.	Infant, neonatal, and post-neonatal mortality: all causes and respiratory causes; SIDS	PM_10_, SO_2_, SO_4_^(2-)^, CO, and non-sulfate PM_10_	Logistic regression	Confounders: mother’s smoking, education, marital status, and race; month of birth; and county average heating degree days Stratified by age (neonatal and post-neonatal), by birth weight (normal and low [<2500g]), and by specific causes within these categories.	No significant association.
Ha et al., 2003 [31]	Time series. 1995–1999, Seoul, South Korea.	1045 post-neonatal deaths.	Post-neonatal mortality: all causes and respiratory causes	PM_10_, SO_2_, CO, O_3_, and NO_2_	Generalized additive Poisson models	Confounders: seasonality, temperature, relative humidity, day of week.	Significant association between short term exposure to PM_10_ and risk of post-neonates and specially with that of respiratory mortality.
Romieu et al., 2004 [18]	Case-crossover. 1997-2001, Ciudad Juarez, Mexico.	628 post-neonatal deaths.	Post-neonatal mortality: all causes and respiratory causes	PM_10_	Conditional logistic regression	Confounders: Temperature and season, Stratified by socioeconomic index.	No significant association had been revealed.
Dales et al., 2004 [19]	Time-series. 1984–1999, 12 Canadian cities.	1556 SIDS.	SIDS	SO_2_, CO, O_3_, PM_10_, PM_2.5_, and NO_2_	Random-effect regression model	Confounders: Adjustments for season alone or the combination of daily mean temperature, relative humidity, and changes in barometric pressure.	Significant association between short-term exposure to NO_2_ and increased rates of SIDS.
Lin et al., 2004 [20]	Time series. 1998–2000, Sao Paulo.	6696 neonatal deaths.	Neonatal mortality	PM_10_, SO_2_, CO, O_3_, and NO_2_	Generalized additive Poisson regression models	Confounders: long- and short- term trend, temperature, humidity, holidays.	Significant association between short-term exposure to PM_10_ exposure and neonatal deaths.
Klonoff-Cohen et al., 2005 [17]	Case control. 1988–1992, Southern California, US.	169 SIDS cases.	SIDS	CO and NO2	Conditional logistic regression	Confounders: postnatal smoking by all live-in household members, low infant birth weight, infant medical conditions at birth, and maternal education.	Significant association between increased risk of SIDS and both short- and long-term exposure to NO_2_.
Yang et al., 2006 [29]	Case-crossover. 1994–2000, Taipei, Taiwan.	471 post-neonatal deaths.	Post-neonatal mortality	PM_10_, SO_2_, CO, O_3_, and NO_2_	Conditional logistic regression	Confounders: temperature; humidity.	A positive but non-significant association between post-neonatal mortality and short term exposure of PM_10_ and NO_2_.
Darrow et al., 2006 [14]	Birth cohort. 1999–2002, US counties.	453 post-neonatal infant respiratory deaths.	Post-neonatal mortality due to respiratory causes	PM_10_, PM_2.5_, and CO	Logistic generalized estimating equations	Confounders: maternal education, marital status, age, primiparity, maternal smoking, county-level poverty indicators, birth region, birth month, and birth year.	A statically significant increased risk of post-neonatal respiratory mortality with long-term exposure to PM_10_ and PM_2.5_.
Ritz et al., 2006 [15]	Case control. 1989–2000, South Coast Air Basin of California, US.	13,146 post-neonatal infants.	Post-neonatal mortality: all causes and due to respiratory causes, SIDS	PM_10_, CO, O_3_, and NO_2_	Conditional logistic regression	Confounders: gender; maternal age; race; education, parental care, season, birth county; parity Stratify by birth weight; gestational age.	A significant association between long-term exposure to NO_2_ and increased risk of post-neonatal mortality. A significant association between both short- and long-term exposure to PM_10_ and all post-neonatal mortality.
Woodruff et al., 2006 [16]	Case control. 1999–2000, California, US.	788 post-neonatal deaths.	Post-neonatal mortality: all causes and respiratory causes; SIDS; external causes of death	PM_2.5_	Conditional logistic regression	Confounders: maternal race, marital status, parity, maternal education, and maternal age. Stratified by birth weight and gestational age.	A significant association between post-neonatal mortality from respiratory causes and long-term exposure to PM_2.5_.
Tsai et al., 2006 [30]	Case-crossover. 1994–2000, Kaohsiung, Taiwan. (Industrial city).	207 post-neonatal deaths.	Post-neonatal mortality	PM_10_, SO_2_, CO, O_3_, and NO_2_	Conditional logistic regression	Confounders: Temperature; humidity.	Positive but no significant association between the risk of post-neonatal deaths with daily concentration for PM_10_ and NO_2_.
Hajat et al., 2007 [9]	Time-series. 1990–2000, UK.	22,288 total Infant deaths.	Infant Mortality, neonatal, and post-neonatal mortality	PM_10_, SO_2_, CO, O_3_, NO_2_, and NO	Poisson generalized linear models;	Confounders: influenza A, respiratory syncytial virus activity, temperature, humidity, secular trends, seasonal fluctuations.	No significant association between short-term exposure of PM_10_ and NO_2_ and all infant, neonatal, and post-neonatal mortality.
Son et al., 2008 [28]	Case crossover and Time-series analysis 1993–2003, Seoul, Korea.	766 post-neonatal deaths.	Post-neonatal mortality	PM_10_, SO_2_, CO, O_3_, and NO_2_	Conditional Logistic Regression; Generalized additive models	Confounders: temperature; humidity; air pressure.	No significant association between PM_10_ exposure and post-neonatal mortality. Positive associations between NO_2_ exposure and post-neonatal mortality, but not statistically significant.
Woodruff et al., 2008 [13]	Birth cohort study. 1999–2002, 96 US counties.	6639 post-neonatal deaths.	Post-neonatal mortality: all causes and respiratory causes; SIDS	PM_10_, PM_2.5_, SO_2_, CO, and O_3_	Logistic regression incorporating generalized estimating equations	Confounders: maternal factors (race, marital status, education, age, and prim-parity), percentage of county population below poverty, region, birth month, birth year.	A significant statically increase of risk of only respiratory-related post-neonatal mortality for a 10 µg/m3 increase in PM_10_.
Carbajal-Arroyo et al., 2011 [8]	Case-crossover. 1997–2005. Mexico City Metropolitan Area.	12,079 post-neonatal deaths.	Post-neonatal mortality: all causes and respiratory causes	PM_10_ and O_3_	Conditional Logistic Regression	Confounders: weather conditions and day of the week. Effect modification by socioeconomic status and sex.	The risk of post-neonatal mortality all cause and respiratory cause significantly increase with short-term exposure to PM_10_.
Scheers et al., 2011 [25]	Case-crossover. 1998–2006, Flanders, Belgium.	2382 infant deaths.	Infant mortality, early and late neonatal mortality; post-neonatal; All causes and by causes	PM_10_	Conditional Logistic Regression	Confounders: temperature. Stratified by: age groups, maturity (preterm versus term birth), Socio-economic status, and cause of death.	Statically significant increased risk of infant mortality for increased daily mean PM_10_. Stronger and significant association found for late neonates’ mortality.
Son et al., 2011 [27]	Cohort 2004–2007. Seoul Korea.	225 post-neonatal deaths.	Post-neonatal mortality: all causes and respiratory causes; SIDS	TSP, PM_10_, PM_10-2.5_, and PM_2.5_	Extended Cox proportional hazards modeling with time-dependent covariates	Confounders: sex, gestational period, season of birth, maternal age and educational level, and heat index. Stratified by birth weight (normal versus low).	Statistically significant association between long-term exposure to PM and infant mortality from all causes or respiratory causes for normal-birth-weight infants.
Padilla et al., 2013 [24]	Ecological- Spatial 2002–2009, France.	1200 infant deaths.	Infant Mortality	NO_2_	Generalized Additive models	Confounders: neighborhood socioeconomic deprivation.	The spatial excess risk of infant mortality was not explained by spatial variation of NO_2_ concentrations.
Arceo et al., 2016 [10]	Birth and death cohort 1997–2006, Mexico City Metropolitan Area and Guadalajara.	24,691 infant deaths.	Infant and Neonatal Mortality	PM_10_, SO_2_, CO, and O_3_	Regression model: Fixed effect model	Confounders: thermal inversion (instrumental variables), temperature and weather conditions, and municipality-effects.	Statistically significant increased rate in infant mortality for increases in PM_10_ exposure.
Yorifuji et al., 2016 [26]	Case-crossover 2002–2013, Tokyo Metropolitan Government.	2086 infant deaths.	Infant, neonatal Mortality–Post-neonatal mortality: all causes and by separated cause	PM_2.5_; PM_7-2.5_; SPM	Conditional logistic regression	Confounders: daily number of influenza patients; ambient temperature, relative humidity, holidays.	Statically significant association between short-term exposure to PM and infant and post-neonatal mortality.
Padilla et al., 2016 [23]	Ecological Spatial 2002–2009, France.	2464 infant deaths.	Infant and neonatal mortality	NO_2_	Generalized Additive Models	Confounders: None Stratified by times-period.	Results suggest that spatial excess risk of infant and neonatal mortality was largely explained by socioeconomic deprivation index and NO_2_ concentrations.
Heft-Neal et al., 2018 [32]	Cohort 2001–2015, 30 Sub-Saharan African Countries.	About 70,339 infant deaths.	Infant Mortality	PM_2.5_	Fixed-effects regression	Confounders: None Stratified by time period and wealth level. Accounted for time-invariant differences in air pollution and mortality across locations-cluster effect and for year-effect.	Strong and linear association between infant mortality with PM_2.5_ exposure.
Litchfield et al., 2018 [22]	Case-crossover. 1996–2006, UK.	211 cases of SIDS.	SIDS	PM_10_, SO_2_, CO, O_3_, NO_2_, and NO	Conditional Poisson regression	Confounders: temperature; holidays Stratified by levels of household wealth.	Statically significant association between previous day pollutant concentration (NO_2_ and PM_10_) and SIDS.
Gouveia et al., 2018 [12]	Ecological Time series 1997–2005, Mexico city, Santiago Chile, Sao Paulo, and Rio de Janeiro.	8762 Infant deaths.	Infants and children mortality due to respiratory causes	PM_10_ and O_3_	Generalized Additive Models	Confounders: Time trend, seasonality, holidays, temperature; humidity. Stratified by warm/cold season; and for each city.	Results suggest an increase in the percentage of the risk of death due to respiratory diseases in infants for 10µg/m-3 increase in PM_10_.

Legends: PM: Particulate Matter; PM_10_: particulate matter with an aerodynamic diameter up to 10 μm; PM_2.5_: particulate matter with an aerodynamic diameter up to 2.5 μm; TSP: total suspended particulate; SPM: suspended particulate matter NO: nitrogen monoxide; NO_2_: nitrogen dioxide; O_3_: Ozone, SO_2_: sulfure dioxide; CO: Carbon monoxide; SIDS: Sudden infant death syndrome.

**Table 2:** Summary of approaches used to assess the residential exposure measures.

Approach	Level of Exposure Assigned to the Population	Database/Model Used	Pollutants	Indicators	Data Sources of Air Pollution	Authors, Date
Monitoring station-based approach						
Average from all monitoring stations	Country-specific level	27 monitoring stations distributed evenly throughout Seoul.	TSP, PM_10_, PM_10-2.5_, and PM_2.5_	24 h averages	Department of Environment, Republic of Korea	Son et al., 2010 [27]
		27 monitoring stations distributed evenly throughout Seoul.	PM_10_, SO_2_, CO, O_3_, NO_2_	24 h averages for PM_10_, SO_2_, NO_2_ exposure	Department of Environment, Republic of Korea	Son et al., 2010 [28]
	The entire El-Paso/Ciudad Juarez airshed level	Nine Fixed monitoring stations distributed throughout Ciudad Juarez.	PM_10_, O_3_	24 h average for PM10	Ciudad Juarez monitoring network system	Romieu et al., 2004 [18]
	City level	A minimum of two monitoring sites for each city, except for Middlesbrough and Newcastle, where only one site was used.	PM_10_, SO_2_, CO, O_3_, NO_2_, and NO	Daily average	United Kingdom Air Quality Network	Hajat et al., 2007 [9]
		Six air quality monitoring stations in Taipei city.	PM_10_, SO_2_, CO, O_3_, and NO_2_	Daily average	Taiwan Environmental Protection Administration	Yang et al., 2006 [29]
		Six air quality monitoring stations in Kaohsiung city.	PM_10_, SO_2_, CO, O_3_, and NO_2_	Daily average	Taiwan Environmental Protection Administration, a central governmental agency	Tsai et al., 2006 [30]
	Post-code level	10 station across four postal code areas in the West Midlands region.	PM_10_, SO_2_, CO, O_3_, NO_2_, NO_x_, NO	Daily average concentrations	UK air quality archive managed by the Department for the Environment, Food and Rural Affairs	Litchfield et al., 2018 [22]
Average from existing Monitoring stations	County level	A selection of monitoring stations most likely to reflect population exposure.	PM_10_, PM_2.5_, SO_2_, CO, and O_3_	24 h average measured once every 6 days for PM_10_ and PM_2.5_	United States Environmental Protection Agency	Woodruff et al., 2008 [13]
		No information available.	PM_10_, PM_2.5_, and CO	Ambient levels in their county during their first 2 months of life	United States Environmental Protection Agency	Darrow et al., 2006 [14]
		All monitoring station.	PM_10_, SO_2_, SO_4_, CO, and non-sulfate PM_10_	Annual average	The United States Environmental Protection Agency’s Aeromatic Information and Retrieval System	Lipfert et al., 2000 [21]
	Municipality level	One station per municipality or average if more than one station.	PM_10_, O_3_	24 h daily mean for PM_10_ exposure	Metropolitan Area Monitoring Network System.	Carbajal-Arroyo et al., 2011 [8]
	City level	Monitoring stations existing (*When data were available from >1 monitoring site, they were averaged).*	SO_2_, CO, O_3_, PM_10_, PM_2.5_, and NO_2_	24 h average	National Air Pollution Surveillance system	Dales et al., 2004 [19]
		Monitoring stations located within the city.	PM_10_, SO_2_, CO, O_3_, and NO_2_	Daily mean levels	The São Paulo State Sanitary Agency	Lin et al., 2004 [20]
		All monitoring stations in each city (reflecting background air pollution level, not influenced by local sources).	PM_10_, 0_3_	Daily 24 h mean average of PM_10_ Daily 8 h maximum moving average for 0_3_	Secretary of Environment and Natural Resources in Mexico. Local environmental agencies which report to the Ministry of Environment in Brazil. Local governmental networks in Chile.	Gouveia et al., 2018 [12]
Nearest monitoring station	Individual level	The nearest monitor within 5 miles of the mother’s residence *lues were used to identify the nearest monitor within 5 miles of each mother’s residence.*	PM_2.5_	24 h average every 6 days	California Air Resources Board	Woodruff et al., 2006 [16]
	Zip code level	The nearest best air monitoring station within 10 miles of the mother zip code and taking into account 3 additional parameters: distance, geographic features, and wind flow patterns.	PM_10_, CO, O_3_, and NO_2_	Hourly measurements for NO_2_, CO, and O_3_ 24 h average measurements for PM_10_	South Coast Air Quality Management monitoring station, from electronic files assembled by the California Department of Health Services	Ritz et al., 2006 [15]
		The monitoring station closest to the infant address/zip code.	CO and NO_2_	Maximum daily 1 h average	California Air Resources Board	Klonoff-Cohen et al., 2005 [17]
	Municipality level	48 of the 56 municipalities in Mexico cities, located within 15 km of a station Measures of pollution constructed using the inverse of the distance to nearby stations as weight.	PM_10_, SO_2_, CO, O_3_	Maximum daily 8 h average for CO and average over the week Maximum daily 24 h average for PM_10_ and average over the week Weekly averages for SO_2_ and for O_3_	Automatic Network of Atmospheric Monitoring	Arceo et al., 2016 [10]
	Wards level	Monitoring station, named general station, located about 12 km from the central point of the 23 wards.	PM_2.5_; PM_7-2.5_; SPM	Daily concentrations	Bureau of Environment of the Tokyo Metropolitan Government	Yorifuji et al., 2016 [26]
Modeling based approaches						
Modeling approaches	Municipality level	Land use regression model and kriging interpolation model using land cover data obtained from satellite images.	PM_10_	Daily concentrations	Network of automatic monitoring sites	Scheers et al., 2011 [25]
	Census block level	Atmospheric Dispersion Modeling System.	NO_2_	Annual average	Local air quality monitoring networks	Padilla et al., 2013 [24], 2016 [23]
	Country level	Satellite based measurements.	PM_2.5_	Annual average	Atmospheric Composition Analysis Group at Dalhousie University	Heft-Neal et al., 2018 [32]

**Table 3:** Definition and assessment of window of exposure.

	Windows of Exposure	Pollutants	Authors
**Short term exposure**			
Daily exposure	The day of the death (Lag 0)	PM_10_, O_3_	Carbajal-Arroyo et al., 2011 [8]
		PM_2.5_; PM_7-2.5_; SPM	Yorifuji et al., 2016 [26]
		PM_10_	Scheers et al., 2011 [25]
		PM_10_, SO_2_, CO, O_3_, and NO_2_	Lin et al., 2004 [20]
		PM_10_, SO_2_, CO, O_3_, and NO_2_	Ha et al., 2003 [31]
	The day before death (Lag 1)	PM_10_, SO_2_, CO, O_3_, NO_2_ NO	Litchfield et al., 2018 [22]
		PM_10_	Romieu et al., 2004 [18]
		PM_10_, O_3_	Carbajal-Arroyo et al., 2011 [8]
	Two days before death (Lag 2)	PM_10_, O_3_	Carbajal-Arroyo et al., 2011 (11) [8]
		PM_10_, SO_2_, CO, O_3_, NO_2_ NO	Litchfield et al., 2018 [22]
		PM_10_	Romieu et al., 2004 [18]
	Three days before death (Lag3)	PM_10_, SO_2_, CO, O_3_, NO_2_ NO	Litchfield et al., 2018 [22]
	Four days before death (Lag4)	PM_10_, SO_2_, CO, O_3_, NO_2_ NO	Litchfield et al., 2018 [22]
	Five days before death (Lag5)	PM_10_, SO_2_, CO, O_3_, NO_2_ NO	Litchfield et al., 2018 [22]
	Six days before death (Lag6)	PM_10_, SO_2_, CO, O_3_, NO_2_ NO	Litchfield et al., 2018 [22]
Cumulative Exposure	Over 2 days before death (Lag 0-2)	PM_10_, SO_2_, CO, O_3_, NO_2_, and NO	Hajat et al., 2007 [9]
		PM_10_, SO_2_, CO, O_3_, and NO_2_	Yang et al., 2006 [29]
		PM_10_, SO_2_, CO, O_3_, and NO_2_	Tsai et al., 2006 [30]
		PM_10_	Romieu et al., 2004 [18]
		PM_10_, SO_2_, CO, O_3_, NO_2_ NO	Litchfield et al., 2018 [22]
	Over 3 days before the death (Lag 0-3)	PM_10_ O_3_	Gouveia et al., 2018 [12]
		SO_2_, CO, O_3_, PM_10_, PM_2.5_, NO_2_	Dales et al., 2004 [19]
		PM_10_	Romieu et al., 2004 [18]
		PM_10_, O_3_	Carbajal-Arroyo et al., 2011 [8]
		CO and NO_2_	Klonoff-Cohen et al., 2005 [17]
	Over 4 days before the death (Lag 0-4)	PM_2.5_; PM_7-2.5_; SPM	Yorifuji et al., 2016 [26]
	Over 6 days before the death (Lag 0-6)	PM_10_, SO_2_, CO, O_3_, NO_2_ NO	Litchfield et al., 2018 [22]
	Over 7 days before the death (Lag0-7)	CO and NO_2_	Klonoff-Cohen et al., 2005 [17]
	Over two to seven days before death (Lag 2-7)	PM_10_, SO_2_, CO, O_3_, and NO_2_	Lin et al., 2004 [20]
**Long term exposure**			
Cumulative Exposure	Weekly exposure	PM_10_, SO_2_, CO, O_3_	Arceo et al., 2016 [10]
	2 weeks before death	PM_10_, CO, O_3_, and NO_2_	Ritz et al., 2006 [15]
	1 month before death (or 30 days)	PM_10_, CO, O_3_, and NO_2_	Ritz et al., 2006 [15]
		CO and NO_2_	Klonoff-Cohen et al., 2005 [17]
	The first 2 months of life	PM_10_, PM_2.5_, SO_2_, CO, and O_3_	Woodruff et al., 2008 [13]
		PM_10_, PM_2.5_, and CO	Darrow et al., 2006 [14]
	2 months before death	PM_10_, CO, O_3_, and NO_2_	Ritz et al., 2006 [15]
	6 months before death	PM_10_, CO, O_3_, and NO_2_	Ritz et al., 2006 [15]
	Between birth and the death	PM_2.5_	Woodruff et al., 2006 [16]
		PM_2.5_	Heft-Neal et al., 2018 [32]
		TSP, PM_10_, PM_10-2.5_, and PM_2.5_	Son et al., 2010 [27]
		CO and NO_2_	Klonoff-Cohen et al., 2005 [17]
	By trimester of pregnancy	TSP, PM_10_, PM_10-2.5_, and PM_2.5_	Son et al., 2010 [27]
	During the 9 months of pregnancy	TSP, PM_10_, PM_10-2.5_, and PM_2.5_	Son et al., 2010 [27]
		PM_2.5_	Heft-Neal et al., 2018 [32]
No specific window of exposure		PM_10_, SO_2_, SO_4_^(2-)^, CO, and non-sulfate	Lipfert et al., 2000 [21]
		NO_2_	Padilla et al., 2013 [24], 2016 [23]

**Table 4:** Begg’s test on the effect of air pollutants on infant mortality.

Birth Outcomes	Pollutants	N *	*p*-Value **
POST-NEONATAL DEATH ALL-CAUSES	NO_2_ long term exposure	5	≈1
	PM_10_ long term exposure	9	0.23
	PM_2.5_ long-term exposure	4	0.042 ***
RESPIRATORY POST-NEONATAL DEATH	PM_10_ long- and short-term exposure	8	0.32
	PM_10_ long-term exposure	4	0.042 ***
SUDDEN INFANT DEATH SYNDROME	PM_2.5_ long- and short-term exposure	4	0.49
	PM_10_ long- and short-term exposure	5	0.62

*: number of studies. **: *p*-value resulting from the Begg’s rank test, *** significant *p*-value (<0.05).

## Supplementary Materials

The following are available online at https://www.mdpi.com/1660-4601/17/8/2623/s1, Figure S1: funel plot, Table S1: Definitions of Infant mortality outcomes and studied population (order by outcome), Table S2: Definitions of Infant mortality outcomes and measures of association for meta-analysis, Table S3: Sensitivity analysis, Table S4: Characteristics of the included studies in meta-analysis: the scores for each criterion and the quality index, S1: Text. Quality effect model methods.
