# Supplementary material for: Infant Mortality Related to NO2 and PM Exposure: Systematic Review and Meta-Analysis
Source: Int J Environ Res Public Health. 2020 Apr 11;17(8):2623. doi: 10.3390/ijerph17082623 (PMC7215927; doi:10.3390/ijerph17082623)
Supplement: Supplementary file 1 [file ijerph-17-02623-s001.zip › supple/S1.DOCX]

**Supplementary Materials S1:Text. Quality effect model methods**

Individual quality assessment methodology was adapted from Croteau *et al* in 2009 and doi and Thalib in 2008. The checklist was defined by researcher consensus. It assigned a maximum of 1.00 point for the different methodological criteria and a quality score (Qi=$( \frac{\sum_{1}^{10} score criteria}{10}$)) is calculated for each study included in the meta-analysis.

Ten Criteria are defined as follow:

1. **Sample size**

(**1**): completely satisfactory/ justified by power analysis; (**0.5**): somewhat satisfactory; (**0**): not sufficient/not justified.

1. **Design**

(**1**): cohort; (**0.75**): case-crossover; case-control; (**0.5**): ecological ; time serie

1. **Country** where the study was carried out

(**1**): With good working and living conditions/high socio-economic standard; (**0.5**): Difficult conditions/lower socio-economic standard; (**0.25**): Very difficult conditions/very low socio-economic standard; (**0**): Not reported.

(1: USA, UK, Sweden, Latina America; 0.5: China)

1. **Timeframe**

(**1**): Reported; (**0**): Not reported.

1. **Geocodage rate**

(**1**): ≥80%/considerable part of the population; (**0.75**): Not reported.

**6- Definition of infant death**

**-->** (**1**): infant death excluding death due to accident and external causes or specific cause death; (**0.75**): death among singleton birth or among term birth; (**0.5**): overall death.

**7- Assessment of infant death**

(**1**): valid database; (**0.5**): self-report; (**0**): not specified.

1. **Assessment of the exposure**:

(**1**): individual measure; (**0.75**): fine spatial level (zip code, municipality, ward level) (**0.5**): country level

1. **Adjustments for covariates** (cov)

(**1**): At least 1 (cov) in each of the three covariates (baby’s characteristic, mother’s characteristics, or meteorological condition), (**0.75**): At least 1 (cov) baby’s characteristic and at least 1 (cov) mother’s characteristic (or meteorological condition); **0.5**): At least 1 (cov) in meteorological condition; (**0**): no covariates

1. **Effect size calculation for meta-analysis based on odds ratios**

(**1**): no transformations and no data imputation; (**0.75**): mild transformation and no data imputation; (**0.5**): several transformations and no data imputation; (**0.25**): considerable transformations and data imputation
