# Supplementary material for: Infant Mortality Related to NO2 and PM Exposure: Systematic Review and Meta-Analysis
Source: Int J Environ Res Public Health. 2020 Apr 11;17(8):2623. doi: 10.3390/ijerph17082623 (PMC7215927; doi:10.3390/ijerph17082623)
Supplement: Supplementary file 1 [file ijerph-17-02623-s001.zip › supple/Supplementary Table S1.docx]

**Supplementary Table S1.** Definitions of Infant mortality outcomes and studied population (order by outcome).

|  | **Type(s) or subtype(s)** | **Outcome classification** | **Population study** | **Database study** | **Authors, date** |
| --- | --- | --- | --- | --- | --- |
| **Infant mortality** |  |  |  |  |  |
|  | Overall death | Death >1 year old | All birth between 2001 and 2015 (990,696 births) | 65 available Demographic and Health Surveys across 30 sub-Saharan African countries | **Heft-Neal et al., 2018** [32] |
|  |  |  | 2,464 infant deaths | Death certificate in the city hall of each metropolitan area. | **Padilla et al., 2016** [23] |
|  |  |  | All infants (under 1 year old) who died between January  2002 and December 2013 (2,086 infants) | Ministry of health, labour and Welfare in Japan | **Yorifuji et al., 2016** [26] |
|  |  |  | All infant death  (24,691 infant deaths) | Death and birth certificates from the Ministry of Health (Secretarıa de Salud Publica) | **Arceo et al., 2016** [10] |
|  |  |  | 1,200 infant deaths | Death certificate in the city hall of each metropolitan area. | **Padilla et al., 2013** [24] |
|  |  |  | All infant deaths  2,382 infant deaths | Flemish Agency for Care and Health (Brussels, Belgium). | **Scheers et al., 2011** [25] |
|  |  |  | All infant deaths  22,288 Infant deaths | Office for National Statistics for the 10 major cities of the study. | **Hajat et al., 2007** [9] |
|  |  |  | All infants born alive (excluded multiple births, deaths attributable to external causes)  164,161 births | California Department of Health Services. | **Ritz et al., 2006** [15] |
|  |  |  | All births  1,443,768 births | National linked Birth/infant death dataset (NLBDDS) from the National Center for Health Statistics. | **Lipfert et al., 2000**  [21] |
|  | Respiratory cause |  | All children death  8,762 infant death | Ministry of health for Rio de Janeiro (Brazil) and Santiago (Chile), Mortality information System of Sao Paulo, National Institute of Statistics and Geography (INEGI) for Mexico city and further checked at the National Institute of Public Health (INSP). | **Gouveia et al., 2018** [12] |
|  |  | Death related to respiratory diseases: ICD 10^th^ revision codes J00-J99 | 2,086 infants | Ministry of health, labour and Welfare in Japan | **Yorifuji et al., 2016** [26] |
|  |  | Two different definitions: The traditional definition, (ICD) 460-519, involves only pneumonia and influenza for infants; ICD 769 (respiratory distress) and ICD 770 (other respiratory conditions of the newborn) | 1,443,768 births | Death and birth certificates linked from the National Center for Health Statistics.  National linked Birth/infant death dataset (NLBDDS) | **Lipfert et al., 2000** [21] |
|  | Cardiac death | Death related to cardiac diseases: ICD 10^th^ revision codes I10-I99 | 2,086 infants | Ministry of health, labour and Welfare in Japan | **Yorifuji et al., 2016** [26] |
|  | Cardio-resipratory | Respiratory causes includes respiratory and cardiovascular disease, NC | All infant death  24,691 infant deaths | Death and birth certificates from the Ministry of Health (Secretarıa de Salud Publica) | **Arceo et al., 2016** [10] |
|  |  | Cardiorespiratory diseases (ICD-10 code) (I00–J99) | All infant deaths  2,382 infant deaths | Flemish Agency for Care and Health (Brussels, Belgium). | **Scheers et al., 2011** [25] |
|  | Congenital and chromosomal abnormalities | Death related to congenital and chromosomal abnormalities: ICD 10^th^ revision codes Q00-Q99 | 2,086 infants | Ministry of health, labour and Welfare in Japan | **Yorifuji et al., 2016** [26] |
|  |  | Congenital and chromosomal abnormalities (ICD-10 code) (Q00–Q99) | All infant deaths  2,382 infant deaths | Flemish Agency for Care and Health (Brussels, Belgium). | **Scheers et al., 2011** [25] |
|  | Perinatal circumstances | CD 10^th^ revision codes P00-P96 | 2,086 infants | Ministry of health, labour and Welfare in Japan | **Yorifuji et al., 2016** [26] |
|  |  | (ICD-10 code) (P00–P96) | All infant deaths  2,382 infant deaths | Flemish Agency for Care and Health (Brussels, Belgium). | **Scheers et al., 2011**[25] |
|  | External causes | Deaths from accidents and homicides; internal cause encompasses all causes not including accidents or homicides, NC | All infant death  24,691 infant deaths | Death and birth certificates from the Ministry of Health (Secretarıa de Salud Publica) | **Arceo et al., 2016** [10] |
|  | Non-respiratory causes | Digestive, congenital, accidents, homicides etc. , NC | All infant death  24,691 infant deaths | Death and birth certificates from the Ministry of Health (Secretarıa de Salud Publica) | **Arceo et al., 2016** [10] |
| **Neonatal mortality** |  |  |  |  |  |
|  | Overall death | Death <28 deays old | All infant deaths  (2,464 infant deaths) | Death certificate in the city hall of each metropolitan area. | **Padilla et al., 2016** [23] |
|  |  |  | 2,086 infants | Ministry of health, labour and Welfare in Japan | **Yorifuji et al., 2016** [26] |
|  |  |  | All infant death  24,691 infant deaths | Death and birth certificates from the Ministry of Health (Secretarıa de Salud Publica) | **Arceo et al., 2016** [10] |
|  |  |  | All infant deaths  2,382 infant deaths | Flemish Agency for Care and Health (Brussels, Belgium). | **Scheers et al., 2011** [25] |
|  |  |  | All infant deaths  22,288 Infant deaths | Office for National Statistics for the 10 major cities of the study. | **Hajat et al., 2007** [9] |
|  |  |  | All births  1,439,089 births | Death and birth certificates linked from the National Center for Health Statistics.  National linked Birth/infant death dataset (NLBDDS) | **Lipfert et al., 2000** [21] |
|  |  |  | All infants born alive (excluded multiple births, deaths attributable to external causes)  164,161 births | California Department of Health Services. | **Ritz et al., 2006** [15] |
|  | Respiratory causes | Two different definitions: The traditional definition, (ICD) 460-519, involves only pneumonia and influenza for infants; ICD 769 (respiratory distress) and ICD 770 (other respiratory conditions of the newborn) | 1,443,768 births | Death and birth certificates linked from the National Center for Health Statistics.  National linked Birth/infant death dataset (NLBDDS) | **Lipfert et al., 2000** [21] |
|  |  | ICD 9^th^ revision codes 460–519, 769, 770.4, 770.7, 770.8, 770.9 + ICD 10^th^ revision codes J00–J98, P22.0, P22.9, P27.1, P27.9, P28.0, P28.4, P28.5, and P28.9 | All infants born alive (excluded multiple births, deaths attributable to external causes)  164,161 births | California Department of Health Services. | **Ritz et al., 2006** [15] |
|  | Cardiorespiratory causes | Respiratory causes (RC) includes respiratory and cardiovascular disease, NC | All infant death  24,691 infant deaths | Death and birth certificates from the Ministry of Health (Secretarıa de Salud Publica) | **Arceo et al., 2016** [10] |
|  |  | Cardiorespiratory diseases (ICD-10 code) (I00–J99) | All infant deaths  2,382 infant deaths | Flemish Agency for Care and Health (Brussels, Belgium). | **Scheers et al., 2011** [25] |
|  | External causes | All deaths from accidents and homicides; internal cause encompasses all causes not including accidents or homicides, NC | All infant death  24,691 infant deaths | Death and birth certificates from the Ministry of Health (Secretarıa de Salud Publica) | **Arceo et al., 2016** [10] |
|  | Congenital and chromosomal abnormalities | (ICD-10 code) (Q00–Q99) | All infant deaths  2,382 infant deaths | Flemish Agency for Care and Health (Brussels, Belgium). | **Scheers, 2011** [25] |
|  | Non-respiratory causes | Digestive, congenital, accidents, homicides etc, NC | All infant death  24,691 infant deaths | Death and birth certificates from the Ministry of Health (Secretarıa de Salud Publica) | **Arceo, 2016** [10] |
|  | Perinatal circumstances | (ICD-10 code) (P00–P96) | All infant deaths  2,382 infant deaths | Flemish Agency for Care and Health (Brussels, Belgium). | **Scheers, 2011** [25] |
| **Post-neonatal mortality** |  |  |  |  |  |
|  | Overall death | Death between 28 days and 1 year | 2,086 infants | Ministry of health, labour and Welfare in Japan | **Yorifuji et al., 2016** [26] |
|  |  |  | All infant deaths  2,382 infant deaths | Flemish Agency for Care and Health (Brussels, Belgium). | **Scheers et al., 2011** [25] |
|  |  |  | All infant deaths  22,288 Infant deaths | Office for National Statistics for the 10 major cities of the study. | **Hajat et al., 2007** [9] |
|  |  |  | All births  1,435,406 births | Death and birth certificates linked from the National Center for Health Statistics.  National linked Birth/infant death dataset (NLBDDS) | **Lipfert et al., 2000** [21] |
|  |  |  | All infant death (excluding  accidental death)  12,079 post-neonatal deaths | Mexico's National Institute of Statistics, Geographic and Informatics and were reviewed for consistency at Mexico's National Institute of Public Health. | **Carbajal-Arroyo et al., 2011** [8] |
|  |  |  | All births with 37–44 weeks of gestation 359,459 births | Korean National Statistical Office. | **Son et al., 2010** [27] |
|  |  |  | All infant deaths  6,107 post-neonatal deaths | Korean National Statistical Office. | **Son et al., 2008** [28] |
|  |  |  | All singleton births with known birth  order, known maternal race, known maternal  education, known marital status, known  maternal age, known birth weight, and a  reported gestational age of up to 44 weeks. 3,583,495 births  6639 post-neonatal | Death and birth certificates linked from the National Center for Health Statistics. | **Woodruff et al., 2008** [13] |
|  |  |  | All infant deaths (*excluded Deaths due to accidents (ICD-9 codes 800–999) and deaths occurring*  *outside of the city were excluded from the analysis)*  471 post-neonatal deaths | Department of Health in charge of the death registration system in Taiwan | **Yang et al., 2006** [29] |
|  |  |  |  |  |  |
|  |  |  | All singleton  births  3877 births/  788 deaths | California Department of Health Services. | **Woodruff et al., 2006** [16] |
|  |  |  | All post-neonatal deaths  (Deaths due to accidents)  207 post-neonatal deaths. | Department of Health in charge of the death registration system in Taiwan | **Tsai et al., 2006** [30] |
|  |  |  | All post-neonatal deaths  628 post-neonatal deaths | death certificate collected from the Ministry  of Health in the state of Chihuahua | **Romieu, 2004 (27)** [18] |
|  |  |  | All infant deaths  6,696 neonatal deaths | The Municipal Mortality information Improvement Program | **Lin, 2004 (30)** [20] |
|  |  |  | All infant death (excluded accidental deaths)  1,045 post-neonatal deaths | Korean National Statistical Office | **Ha, 2003 (32)** [31] |
|  |  |  | All infants born alive (excluded multiple births, deaths attributable to external causes)  164,161 births | California Department of Health Services. | **Ritz et al., 2006** [15] |
|  | Respiratory cause | For 1997: ICD 9^th^ revision codes 460-519  For 1998 – 2005: ICD 10^th^ revision codes J00-J99 | All infant death  12,079 post-neonatal deaths | Mexico's National Institute of Statistics, Geographic and Informatics and were reviewed for consistency at Mexico's National Institute of Public Health. | **Carbajal-Arroyo et al., 2011** [8] |
|  |  | ICD, 10th Revision  codes of J000 –J984 or P271 | All births  2,469,164 births  (453 Postneonatal) | National Center for Health Statistics. | **Darrow et al., 2006** [14] |
|  |  | ICD 10^th^ revision codes J00-J99 | All births with 37–44 weeks of gestation 359,459 births | Korean National Statistical Office. | **Son et al., 2010** [27] |
|  |  | ICD 10^th^ revision codes J00-J99 + code P27.1 [bronchopulmonary dysplasia] | All singleton births with known birth order, known maternal race, known maternal, education, known marital status, known maternal age, known birth weight, and a reported gestational age of up to 44 weeks.  3,583,495 births; 576 deaths | Death and birth certificates linked from the National Center for Health Statistics. | **Woodruff et al., 2008** [13] |
|  |  | ICD 10^th^ revision codes J00–J99 + P27.100–J98 minus codes J69.0 not likely to be influenced by air pollution. | All singleton births  3877 births/ 51 deaths | California Department of Health Services. | **Woodruff et al., 2006** [16] |
|  |  | ICD 10^th^ revision (no precision on the codes) | All infant death (excluded accidental deaths)  71 post-neonatal deaths | Korean National Statistical Office | **Ha et al., 2003** [31] |
|  |  | ICD 9^th^ revision codes 460-519 and ICD 10^th^ revision codes J00–J99 | All post-neonatal deaths  628 post-neonatal deaths/ 216 respiratory deaths | death certificate collected from the Ministry  of Health in the state of Chihuahua | **Romieu et al., 2004** [18] |
|  |  | ICD 9^th^ revision codes 460–519, 769, 770.4, 770.7, 770.8, 770.9 + ICD 10^th^ revision codes J00–J98, P22.0, P22.9, P27.1, P27.9, P28.0, P28.4, P28.5, and P28.9 | All infants born alive (excluded multiple births, deaths attributable to external causes)  164,161 births | California Department of Health Services. | **Ritz et al., 2006** [15] |
|  |  | (ICD-10 code) (I00–J99) | All infant deaths  2,382 infant deaths | Flemish Agency for Care and Health (Brussels, Belgium). | **Scheers et al., 2011** [25] |
|  | External causes | (ICD-10 codes V01Y98) | All singleton births  3877 births/ 55 deaths | California Department of Health Services. | **Woodruff et al., 2006** [16] |
|  | Congenital and chromosomal abnormalities | (ICD-10 code) (Q00–Q99) | All infant deaths  2,382 infant deaths | Flemish Agency for Care and Health (Brussels, Belgium). | **Scheers et al., 2011** [25] |
|  | Perinatal circumstances | (ICD-10 code) (P00–P96) | All infant deaths  2,382 infant deaths | Flemish Agency for Care and Health (Brussels, Belgium). | **Scheers et al., 2011** [25] |
| SIDS |  | (ICD)-9: 798.0 or ICD-10: R95) | Total births,  211 SIDS | Perinatal Institute and Office of National Statistics | **Litchfield et al., 2018** [22] |
|  |  | ICD-10:R95 | 2,086 infants | Ministry of health, labour and Welfare in Japan | **Yorifuji et al., 2016** [26] |
|  |  | ICD 10^th^ revision code R95 | All births with 37–44 weeks of gestation 359,459 births | Korean National Statistical Office. | **Son et al., 2010** [27] |
|  |  | ICD 10^th^ revision codes R95 + R99 (other ill-defined death) | All singleton births with known birth  order, known maternal race, known maternal education, known marital status, known maternal age, known birth weight, and a reported gestational age of up to 44 weeks.  (3,583,495 births; 1,379 SIDS) | Death and birth certificates linked from the National Center for Health Statistics. | **Woodruff et al., 2008** [13] |
|  |  | ICD-10 code R95 | All singleton births  3877 births/ 136 SIDS | California Department of Health Services. | **Woodruff et al., 2006** [16] |
|  |  | SIDS confirmed by postmortem examination | 338 births ; 169 cases | Health department in Southern California. | **Klonoff-Cohen et al., 2005** [17] |
|  |  | ICD-10:R95 | All infant deaths  2,382 infant deaths | Flemish Agency for Care and Health (Brussels, Belgium). | **Scheers et al., 2011** [25] |
|  |  | A sudden and unexplained death of a child lower than 1 year of age for which a clinical investigation and autopsy fail to reveal a cause of death, NC | All births  1,556 SIDS | Statistics Canada Vital Statistics. | **Dales et al., 2004** [19] |
|  |  | ICD-9 code 798.0 and ICD-10 code R95. | All infants born alive (excluded multiple births, deaths attributable to external causes)  164,161 births | California Department of Health Services. | **Ritz et al., 2006** [15] |
|  |  | unexplained deaths, NC | All births  1,443,768 births | National linked Birth/infant death dataset (NLBDDS) from the National Center for Health Statistics. | **Lipfert et al., 2000** [21] |
