# Supplementary material for: Infant Mortality Related to NO2 and PM Exposure: Systematic Review and Meta-Analysis
Source: Int J Environ Res Public Health. 2020 Apr 11;17(8):2623. doi: 10.3390/ijerph17082623 (PMC7215927; doi:10.3390/ijerph17082623)
Supplement: Supplementary file 1 [file ijerph-17-02623-s001.zip › supple/supplementary_Figure S1.docx]

A - Post-neonatal death all-causes and NO_2_

B - Post-neonatal death all-causes and PM_10_

C - Respiratory post-neonatal death and PM_10_

long- and short-term exposure

D - Respiratory post-neonatal death and PM_10_

long-term exposure

E- Respiratory post-neonatal death and PM_2.5_

long-term exposure

F- Sudden Infant Death Syndrome and PM_2.5_

long- and short-term exposure

G- Sudden Infant Death Syndrome and PM_10_

long- and short-term exposure

Figure S1: funel plot
