# Supplementary material for: Infant Mortality Related to NO2 and PM Exposure: Systematic Review and Meta-Analysis
Source: Int J Environ Res Public Health. 2020 Apr 11;17(8):2623. doi: 10.3390/ijerph17082623 (PMC7215927; doi:10.3390/ijerph17082623)
Supplement: Supplementary file 1 [file ijerph-17-02623-s001.zip › supple/supplementary_Table S3.docx]

**Table S3: Sensitivity analysis**

**Post-neonatal death all-causes and NO_2_**

| Study omitted | Odds Ratio | 95% Confidence Interval | |
| --- | --- | --- | --- |
| Son, 2008 | 1.00 | 0.99 | 1.02 |
| Ha, 2003 | 1.01 | 0.98 | 1.04 |
| Hajat, 2007 | 1.00 | 0.99 | 1.02 |
| Yang, 2006 | 1.00 | 0.99 | 1.02 |
| Tsai, 2006 | 1.00 | 0.99 | 1.02 |
| Pooled estimate | 1.00 | 0.99 | 1.02 |

**Post-neonatal death all-causes and PM_10_**

| Study omitted | Odds Ratio | 95% Confidence Interval | |
| --- | --- | --- | --- |
| Scheers, 2011 | 1.01 | 1.00 | 1.03 |
| Carbajal-arroyo, 2011 | 1.01 | 1.00 | 1.03 |
| Son, 2008 | 1.02 | 1.00 | 1.03 |
| Ha, 2003 | 1.01 | 1.00 | 1.02 |
| Romieu, 2004 | 1.01 | 1.00 | 1.03 |
| Hajat, 2007 | 1.01 | 1.00 | 1.03 |
| Carbajal-arroyo, 2011 | 1.01 | 1.00 | 1.03 |
| Yang, 2006 | 1.01 | 1.00 | 1.02 |
| Tsai, 2006 | 1.01 | 1.00 | 1.02 |
| Pooled estimate | 1.01 | 1.00 | 1.02 |

**Respiratory post-neonatal death and PM_10_ long- and short-term exposure**

| Study omitted | Odds Ratio | 95% Confidence Interval | |
| --- | --- | --- | --- |
| Carbajal-arroyo, 2011 | 1.10 | 1.01 | 1.19 |
| Ha, 2003 | 1.06 | 1.00 | 1.12 |
| Scheers, 2011 | 1.09 | 1.01 | 1.18 |
| Romieu, 2004 | 1.10 | 1.02 | 1.20 |
| Darrow, 2006 | 1.07 | 0.99 | 1.16 |
| Son, 2010 | 1.08 | 1.00 | 1.16 |
| Woodruff, 2008 | 1.07 | 0.99 | 1.16 |
| Ritz, 2006 | 1.09 | 0.99 | 1.19 |
| Pooled estimate | 1.08 | 1.01 | 1.17 |

**Sudden Infant Death Syndrome and PM_10_ long- and short-term exposure**

| Study omitted | Odds Ratio | [95%CI] |  |
| --- | --- | --- | --- |
| Scheers, 2011 | 1.05 | 1.01 | 1.09 |
| Litchfield, 2018 | 1.02 | 0.99 | 1.06 |
| Son, 2010 | 1.04 | 1.01 | 1.08 |
| Woodruff, 2008 | 1.05 | 1.01 | 1.08 |
| Ritz, 2006 | 1.07 | 1.01 | 1.12 |
| Pooled estimate | 1.04 | 1.01 | 1.08 |
