# Supplementary material for: Infant Mortality Related to NO2 and PM Exposure: Systematic Review and Meta-Analysis
Source: Int J Environ Res Public Health. 2020 Apr 11;17(8):2623. doi: 10.3390/ijerph17082623 (PMC7215927; doi:10.3390/ijerph17082623)
Supplement: Supplementary file 1 [file ijerph-17-02623-s001.zip › supple/Supplementary_table_S2.docx]

**Supplementary Table S2.** Definitions of Infant mortality outcomes and measures of association for meta-analysis.

|  | **Type(s) or subtype(s)** | **Outcome classification** | **Authors, date** | **Measure of association** |
| --- | --- | --- | --- | --- |
| **Post-neonatal mortality** |  |  |  |  |
|  | Overall death | Death between 28 days and 1 year | **Yorifuji, 2016** [26] | **PM2.5 10 μg/m^3^ increase:**  **Lag 0 : 1.10 (1.02 ; 1.19)** |
|  |  |  | **Scheers, 2011** [25] | **PM10 10 μg/m^3^ increase:**  **Lag 0: 1.01 ( 0.95;1.07)**  **Data not shown with other lag** |
|  |  |  | **Hajat, 2007** [9] | **Average Lag 0-2 :**  **PM10 10 μg/m^3^ increase: 0.99 ( 0.95;1.03)**  **NO2 10 μg/m^3^ increase: 1 (0.98 ;1.04)** |
|  |  |  | **Carbajal-Arroyo, 2011** [8] | **38.7 μg/m^3^ increase PM10:**  **Lag 0: 1.032 ( 0.987;1.078)**  **Lag 1: 1.055 (1.011;1.102)**  **Lag 2: 1.066 (1.021;1.114)**  **Lag 0-2: 1.063 (1.001;1.132)** |
|  |  |  | **Son , 2010** [27] | **Long term**  **PM10 6.93 μg/m^3^ increase: 1.65 (1.18 ;2.31)**  **PM2.5 3.15 μg/m^3^ increase: 1.53( 1.22 ;1.90)** |
|  |  |  | **Son, 2008** [28] | **Lag 0:**  **1 μg/m^3^ increase PM10: 1 (0.998 ;1.002)**  **1 μg/m^3^ increase NO2: 1.002 (0.994 ;1.009)** |
|  |  |  | **Woodruff, 2008** [13] | **Long term:**  **11 μg/m^3^ increase PM10: 1.04 (0.99;1.1)**  **7 μg/m^3^ increase PM2.5: 1.04 (0.98;1.11)** |
|  |  |  | **Yang, 2006** [29] | **Lag 0-2:**  **30.21 μg/m^3^ increase PM10: 1.031 (0.652;1.630)**  **10.34 ppb NO2 increase: 1.017 (0.691;1.497)** |
|  |  |  | **Woodruff, 2006** [16] | **Long term**  **10 μg/m^3^ increase PM2.5: 1.07 (0.93;1.24)** |
|  |  |  | **Tsai, 2006** [30] | **Lag 0-2:**  **67 μg/m^3^ increase PM10: 1.040 (0.340;3.177)**  **17.84 μg/m^3^ increase ppb increase NO2: 1.018 (0.477;2.171)** |
|  |  |  | **Romieu, 2004** [18] | **Lag 1:**  **20 μg/m^3^ increase PM10: 1.02 (0.94;1.11)**  **Lag 2: PM10 1.03 (0.95;1.12)** |
|  |  |  | **Ha, 2003** [31] | **Lag 0:**  **42.9 μg/m^3^ increase PM10: 1.142 (1.096;1.190)**  **14.9 ppb increase NO2: 1.006 (0.957;1.056)** |
|  |  |  | **Ritz, 2006** [15] | **Long term**  **1 pphm increase NO2: 1.08 (1.04;1.11)**  **10 μg/m^3^ increase PM10: 1.04 (1.01; 1.06)** |
|  | Respiratory cause | For 1997: ICD 9^th^ revision codes 460-519  For 1998 – 2005: ICD 10^th^ revision codes J00-J99 | **Carbajal-Arroyo, 2011** [8] | **38.7 μg/m^3^ increase PM10:**  **Lag 0: 1.029 ( 0.959;1.106)**  **Lag 1: 1.053 (0.981;1.132)**  **Lag 2: 1.098 (1.021;1.180)**  **Lag 0-2: 1.091 (0.985-1.209)** |
|  |  | ICD, 10th Revision  codes of J000 –J984 or P271 | **Darrow, 2006** [14] | **Long term:**  **11 μg/m^3^ PM10 increase: 1.21 (1.07;1.37)**  **7 μg/m^3^ PM2.5 increase: 1.09 (0.89;1.33)** |
|  |  | ICD 10^th^ revision codes J00-J99 | **Son , 2010** [27] | **Long term**  **PM10 6.93 μg/m^3^ increase: 6.2 (1.5 ;25.66)**  **PM2.5 3.15 μg/m^3^ increase: 3.15( 1.26 ;7.85)** |
|  |  | ICD 10^th^ revision codes J00-J99 + code P27.1 [bronchopulmonary dysplasia] | **Woodruff, 2008** [13] | **Long term:**  **11 μg/m^3^ increase PM10: 1.18 (1.06;1.31)**  **7 μg/m^3^ increase PM2.5: 1.11 (0.96;1.29)** |
|  |  | ICD 10^th^ revision codes J00–J99 + P27.100–J98 minus codes J69.0 not likely to be influenced by air pollution. | **Woodruff, 2006** [16] | **Long term:**  **PM2.5 2.13 (1.12 ;4.05)** |
|  |  | ICD 10^th^ revision (no precision on the codes) | **Ha, 2003** [31] | **Lag 0:**  **42.9 μg/m^3^ increase PM10: 2.018 (1.784;2.283)**  **14.9 μg/m^3^ increase NO2: 0.638 (0.429; 0.949** |
|  |  | ICD 9^th^ revision codes 460-519 and ICD 10^th^ revision codes J00–J99 | **Romieu, 2004** [18] | **Lag 1: 20 μg/m^3^ increase PM10: 0.95 (0.83;1.09)**  **Lag 2: PM10 1.04 (0.91; 1.19)** |
|  |  | ICD 9^th^ revision codes 460–519, 769, 770.4, 770.7, 770.8, 770.9 + ICD 10^th^ revision codes J00–J98, P22.0, P22.9, P27.1, P27.9, P28.0, P28.4, P28.5, and P28.9 | **Ritz, 2006** [15] | **Long term:**  **1 pphm increase NO2: 1.06 (0.98;1.14)**  **10 μg/m^3^ PM10 increase: 1.05 (1.01;1.1)** |
|  |  | (ICD-10 code) (I00–J99) | **Scheers, 2011** [25] | **Lag 0 :**  **10 μg/m^3^ increase PM10 : 0.98 (0.76 ;1.26)** |
| Fetal deaths |  |  |  |  |
| SIDS |  | (ICD)-9: 798.0 or ICD-10: R95) | **Litchfield et al., 2018** [22] | **Lag 1:**  **12.5 μg/m^3^ increase PM10: 1.16 (1.06;1.27)**  **19.5 μg/m^3^ increase NO2: 1.16 (1.02;1.31)** |
|  |  | ICD-10:R95 | **Yorifuji, 2016** [26] | **Lag 0 10 μg/m^3^ increase PM2.5: 1.11 (0.89;1.38)** |
|  |  | ICD 10^th^ revision code R95 | **Son , 2010** [27] | **Long term:**  **PM10 6.93 μg/m^3^ increase:** 1.15 (0.38-3.48)  **PM2.5 3.15 μg/m^3^ increase: 1.42( 0.71 ;2.87)** |
|  |  | ICD 10^th^ revision codes R95 + R99 (other ill-defined death) | **Woodruff, 2008** [13] | **Long term:**  **11 μg/m^3^ increase PM10: 1.02 (0.89;1.16)**  **7 μg/m^3^ increase PM2.5: 1.1 (0.86;1.20)** |
|  |  | ICD-10 code R95 | **Woodruff, 2006** [16] | **Long term:**  **10 increase PM2.5 0.82 (0.55;1.23)** |
|  |  | ICD-10:R95 | **Scheers, 2011** [25] | **Lag 0**  **PM10 10 μg/m^3^ increase: 0.99 ( 0.89;1.09)** |
|  |  | ICD-9 code 798.0 and ICD-10 code R95. | **Ritz, 2006** [15] | **Long term:**  **1 pphm increase NO2: 1.15 (1.08;1.23)**  **10 μg/m^3^ increase PM10: 1.03 (0.99;1.08)** |
