# Supplementary material for: Infant Mortality Related to NO2 and PM Exposure: Systematic Review and Meta-Analysis
Source: Int J Environ Res Public Health. 2020 Apr 11;17(8):2623. doi: 10.3390/ijerph17082623 (PMC7215927; doi:10.3390/ijerph17082623)
Supplement: Supplementary file 1 [file ijerph-17-02623-s001.zip › supple/Supplementary_table_S4_quality_score.DOCX]

**Supplementary Table S4. Characteristics of the included studies in meta-analysis : the scores for each criterion and the quality index**

| **Auteurs** | **Ha et al., 2003 [30]** | **Romieu et al., 2004 [17]** | **Yang et al., 2006 [28]** | **Darrow et al., 2006 [13]** | **Ritz et al., 2006 [14]** | **Woodruff et al., 2006 [15]** | **Tsai et al., 2006 [29]** |
| --- | --- | --- | --- | --- | --- | --- | --- |
| **Population size** | 1045 (1) | 628 (1) | 471 (1) | 453 (1) | 13,146 (1) | 788 (1) | 207 (1) |
| **Study design, period location,** | Time series (0,5) | Case-crossover (0,75) | Case-crossover (0,75) | Cohort (1) | Case control (0,75) | Case control (0,75) | Case-crossover (0,75) |
| **Country** | Seoul, South Korea (0,5) | Ciudad Juarez, Mexico (0,5) | Taipei, Taiwan (0,5) | US counties (1) | USA (1) | USA (1) | Kaohsiung, Taiwan (0,5) |
| **Timeframe** | 1995-1999 (1) | 1997-2001 (1) | 1994-2000 (1) | 1999-2002 (1) | 1989-2000 (1) | 1999-2000 (1) | 1994-2000 (1) |
| **Definition of infant death** | All infant death (excluded accidental deaths) (1) | All post-neonatal deaths (0,5) | All infant deaths (*excluded Deaths due to accidents (1)* | Specific death (1) | All infants born alive (excluded multiple births, deaths attributable to external causes) (1) | Overall death among singleton births (0,75) | All infant death (excluded accidental deaths) (1) |
| **Assessment of infant death** | Korean National Statistical Office (1) | death certificate collected from the Ministry of Health in the state of Chihuahua (1) | Department of Health in charge of the death registration system in Taiwan (1) | National Center for Health Statistics (1) | California Department of Health Services (1) | California Department of Health Services (1) | Department of Health in charge of the death registration system in Taiwan (1) |
| **Adjustments for personal covariates** | Confounders: seasonality, temperature, relative humidity, day of week (0,5) | Confounders: Temperature and season (0,5) | Confounders: temperature; humidity (0,5) | Confounders: maternal education, marital status, age, primiparity, maternal smoking, county-level poverty indicators, birth region, birth month, and birth year (0,75) | Confounders: gender; maternal age; race; education, parental care, season, birth county; parity Stratify by birth weight; gestational age (1) | Confounders: maternal race, marital status, parity, maternal education, and maternal age. Stratified by birth weight and gestational age (1) | Confounders: Temperature; humidity (0,5) |
| **Effect size** | no transformation (1) | no transformation (1) | no transformation (1) | no transformation (1) | no transformation (1) | no transformation (1) | no transformation (1) |
| **exposure level** | Administrative areas level (0.75) | country level (0.5) | city level (0.75) | country level (0.5) | ZIP code level (0.75) | Individual level (1) | City level (0.75) |
| **Geocodage rate** | Not reported (0,75) | Not reported (0,75) | Not reported (0,75) | Not reported (0,75) | Not reported (0,75) | Not reported (0,75) | Not reported (0,75) |
| **Quality index (Qi)** | 0,825 | 0,75 | 0,825 | 0,9 | 0,925 | 0,925 | 0,825 |

Table : continued

| **Auteurs** | **Hajat et al., 2007 [8]** | **Son et al., 2008 [27]** | **Woodruff et al., 2008 [12]** | **Carbajal-Arroyo et al., 2011 [7]** | **Scheers et al., 2011 [24]** | **Son et al. , 2011 [26]** | **Yorifuji et al., 2016 [25]** | **Litchfield et al., 2018 [21]** |
| --- | --- | --- | --- | --- | --- | --- | --- | --- |
| **Population size** | 22,288  (1) | 766  (1) | 6,639  (1) | 12,079  (1) | 2,382  (1) | 225  (1) | 2086  (1) | 211  (1) |
| **Study design, period location,** | Time-series (0,5) | Case-crossover (0,75) | Cohort  (1) | Case-crossover (0,75) | Case-crossover (0,75) | Cohort (1) | Case-crossover (0,75) | Case-crossover (0,75) |
| **Country** | UK  (1) | Seoul, Korea (0,5) | USA  (1) | Mexico City  (0,5) | Belgium  (1) | Seoul Korea  (0,5) | Tokyo Metropolitan Government.  (0.5) | UK  (1) |
| **Timeframe** | 1990-2000  (1) | 1993-2003  (1) | 1999-2002  (1) | 1997-2005  (1) | 1998-2006  (1) | 2004-2007  (1) | 2002-2013  (1) | 1996-2006  (1) |
| **Definition of infant death** | All infant deaths  (0,5) | All infant deaths (0,5) | Overall death among singleton births  (0,75) | All infant death (excluded accidental deaths)  (1) | All infant deaths (0,5) | infant death among births with 37–44 weeks of gestation 359,459 births (0,75) | All infant deaths  (0,5) | SIDS  (1) |
| **Assessment of infant death** | Office for National Statistics for the 10 major cities of the study (1) | Korean National Statistical Office (1) | Death and birth certificates linked from the National Center for Health Statistics (1) | Mexico's National Institute of Statistics, Geographic and Informatics and were reviewed for consistency at Mexico's National Institute of Public Health.  (1) | Flemish Agency for Care and Health (Brussels, Belgium) (1) | Korean National Statistical Office  (1) | Ministry of health, labour and Welfare in Japan  (1) | Perinatal Institute and Office of National Statistics (1) |
| **Adjustments for personal covariates** | Confounders: influenza A, respiratory syncytial virus activity, temperature, humidity, secular trends, seasonal fluctuations  (0,5) | Confounders: temperature; humidity; air pressure  (0,5) | Confounders: maternal factors (race, marital status, education, age, and prim-parity), percentage of county population below poverty, region, birth month, birth year  (0,75) | Confounders: weather conditions and day of the week. Effect modification by socioeconomic Status and sex (0,5) | Confounders: temperature. Stratified by: age groups, maturity (preterm versus term birth), Socio-economic status and cause of death  (0,75) | Confounders: sex, gestational period, season of birth, maternal age and educational level, and heat index. Stratified by birth weight (normal versus low) (1) | Confounders: daily number of influenza patients ; ambient temperature, relative humidity, holidays (0,5) | Confounders: temperature; holiday Stratified by levels of household wealth (0,5) |
| **Effect size** | no transformation  (1) | no transformation (1) | no transformation  (1) | no transformation  (1) | no transformation (1) | no transformation  (1) | no transformation (1) | no transformation (1) |
| **exposure level** | City level  (0.75) | Country level  (0.5) | Country level (0.5) | Municipality level  (0.75) | Municipality level (0.75) | Country level (0.5) | wards level (0.75) | Post-code level (0.75) |
| **Geocodage rate** | Not reported  (0,75) | Not reported (0,75) | Not reported (0,75) | Not reported (0,75) | Not reported (0,75) | Not reported (0,75) | Not reported (0,75) | Not reported (0,75) |
| **Quality index (Qi)** | 0,825 | 0,75 | 0,875 | 0,825 | 0,85 | 0,85 | 0,775 | 0,825 |
